# Influence of the Ionic Liquid Type on the Gel Polymer Electrolytes Properties

**DOI:** 10.3390/membranes5040752

**Published:** 2015-11-19

**Authors:** Juan P. Tafur, Florencio Santos, Antonio J. Fernández Romero

**Affiliations:** Grupo de Materiales Avanzados para la Producción y Almacenamiento de Energía (MAPA), Campus de Alfonso XIII, Universidad Politécnica de Cartagena, Cartagena 30203, Murcia, Spain; E-Mails: tafur8652@gmail.com (J.P.T.); fsantoscutillas@gmail.com (F.S.)

**Keywords:** ionic liquid-based Gel Polymer Electrolytes, ionic conductivity, cation transport number, zinc batteries

## Abstract

Gel Polymer Electrolytes (GPEs) composed by ZnTf_2_ salt, poly(vinylidene fluoride-co-hexafluoropropylene) (PVdF-HFP), and different ionic liquids are synthesized using n-methyl-2-pyrrolidone (NMP) as solvent. Three different imidazolium-based ionic liquids containing diverse cations and anions have been explored. Structural and electrical properties of the GPEs varying the ZnTf_2_ concentration are analyzed by ATR-FTIR, DSC, TG, and cyclic voltammetry. Free salt IL-GPEs present distinct behavior because they are influenced by the different IL cations and anions composition. However, inclusion of ZnTf_2_ salt inside the polymers provide GPEs with very similar characteristics, pointing out that ionic transport properties are principally caused by Zn^2+^ and triflate movement. Whatever the IL used, the presence of NMP solvent inside the polymer’s matrix turns out to be a key factor for improving the Zn^2+^ transport inside the GPE due to the interaction between Zn^2+^ cations and carbonyl groups of the NMP. High values of ionic conductivity, low activation energy values, and good voltammetric reversibility obtained regardless of the ionic liquid used enable these GPEs to be applied in Zn batteries. Capacities of 110–120 mAh·g^−1^ have been obtained for Zn/IL-GPE/MnO_2_ batteries discharged at −1 mA·cm^−2^.

## 1. Introduction

Gel polymer electrolytes (GPEs) are materials which are neither solids nor liquids, but hold both the cohesive properties of solids and the diffusive character of liquids. Hence, these electrolytes have become relevant due to their use as excellent substitutes of the liquid electrolytes or as separators in ionic devices including batteries, supercapacitors, fuel cells, *etc.* [1,2]. GPEs can be prepared by trapping liquid electrolytes into different polymer hosts. In this case, the salt provides free-mobile ions which take part in the conduction process, the plasticizing solvent is one of the key factors to take into account the increased conductivity in GPE by ions solvating, and the polymer provides mechanical stability. Hence, the morphology and properties of GPEs will depend on the type and amount of polymer host, salt, and solvent present in the polymer matrix [1,2,3,4,5,6,7,8]. Among the host materials used in GPEs, PVdF-HFP has been widely employed because this is a copolymer with enough crystallinity to maintain a mechanical stability and it is amorphous enough to contain liquid electrolytes [6]. Moreover, due to its high dielectric constant, *ε* = 8.4, PVDF–HFP could be the right host to solvate more salt, contributing to enhancing the electrical conductivity of the polymer electrolyte [9].

Different organic solvent plasticizers, such as dimethyl carbonate (DMC), propylene carbonate (PC), and ethylene carbonate (EC), have been extensively used with a wide range of ILs and salts. Plasticizers improve the flexibility of the polymer matrix when added, *i.e.*, promoting amorphicity due to its high dielectric constants; EC = 90.5 and PC = 60.6 [10].

Moreover, the interaction between solvent C=O groups and salt cations improves the cation transport inside the membrane [11,12,13,14]. Recently, our group has reported an interaction between Zn^2+^ cations and C=O groups of the NMP solvent inside the IL-based GPEs, which has been proven to be fundamental to increase the Zn^2+^ cation transport inside the GPE [14]. Carbonyl groups of NMP, as well as those on plasticizers such as EC and PC, are able to reduce the Zn^2+^ coordination with anions in the GPE, avoiding or reducing the cluster formation. Thus, cationic transport is favored and, hence, higher cationic conductivity results [15].

In addition, inclusion of ionic liquids (ILs) instead of classical salt solutions has helped to improve the properties of the GPEs for supercapacitors and battery applications [3,4,11,12,13,14,16,17,18,19,20,21,22,23,24,25]. Their use is justified by important attributes, including a wide electrochemical window, high ionic conductivity, high thermal stability, non-volatility, non-flammability, *etc.* Furthermore, systems composed by a host, IL, and salt have been announced as relevant to battery technology, where it may lead to enhanced Li^+^, Na^+^, or Zn^2+^ transfer between the electrodes [3,14,24,25,26,27,28,29].

The main aim of this paper is to investigate new GPEs based on ZnTf_2_ and different ILs to be used in Zn batteries. These GPEs have been synthesized using NMP as solvent as it plays an important role enhancing cation transport numbers. However, the inclusion of an IL inside this kind of GPE has also been demonstrated as important to obtain a good polymer electrolyte. Therefore, in this work we have studied the influence of ILs, containing different types of cation and anions, on the electrical and structural properties of these GPEs. We have synthesized GPEs based on PVDF-HFP and ZnTf_2_ salt using three different ionic liquids (Table 1). These GPEs have been studied using several spectroscopic and electrochemical techniques in order to find out whether ion transport is modified or not, depending on the anions and cations type forming the ILs, as well as the extent of this modification.

**Table 1:** Composition of all GPEs synthesized and the nomenclatures used. IL designs the different ionic liquids: EMIM TFSI (ET), BMIM Tf (BTf), and EMIM Tf (ETf). In all cases 3.6 g of NMP was employed. PVdF-HFP polymer is also included.

GPE name	PVdF-HFP/g	IL/g	ZnTf_2_/g
PVdF-HFP	0.5	0	0
IL-GPE	0.5	0.445	0
ZnTf_2_ GPE	0.5	0	0.255
IL-ZnTf_2_ GPE 1	0.5	0.445	0.127
IL-ZnTf_2_ GPE 2	0.5	0.445	0.255
IL-ZnTf_2_ GPE 3	0.5	0.445	0.382
IL-ZnTf_2_ GPE 4	0.5	0.445	0.509
IL-ZnTf_2_ GPE 5	0.5	0.445	0.636
IL-ZnTf_2_ GPE 6	0.5	0.445	0.763

## 2. Results and Discussion

### 2.1. Structural Characterization of the Gel Polymer Electrolytes

#### 2.1.1. XRD

**Figure 1:**
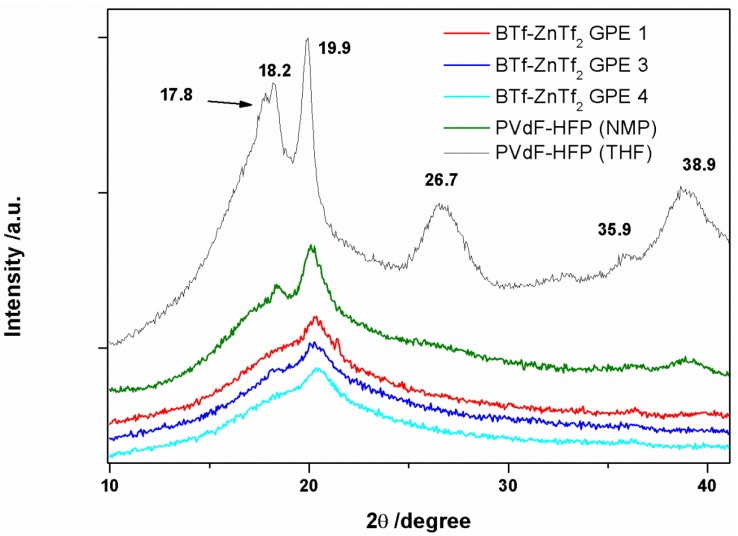
XRD spectra of PVdF-HFP (THF), PVdF-HFP (NMP), and BTf-ZnTf_2_ GPE 1, 3, and 4.

The X-ray diffraction spectroscopy (XRD) is a useful tool to distinguish the different crystalline phases of PVdF-HFP [3,6,14,30,31,32,33,34]. All phases show a big peak at approximately 20°. Both α and γ phases have a second peak at ~18°, whereas the β phase has a broader single line in the same region. However, there is a clear change in the PVDF-HFP spectra when the film is synthesized using THF or NMP as it has been previously reported [14]. This result indicates that NMP modifies the PVdF-HFP structure, which may be related with interactions between NMP molecules and PVdF-HFP chains. Retention of NMP molecules inside the polymer has been confirmed by different ways, as it will be described below. Incorporation of IL and ZnTf_2_ salt into the GPE produced even wider and lower intensity peaks indicating a more amorphous structure, which can be caused by IL and ZnTf_2_ blended with the PVdF-HFP polymer. Figure 1 shows the XRD spectra of PVdF-HFP membrane with BMIM Tf IL at different concentrations of ZnTf_2_, which are very similar to those found for EMIM Tf-based GPEs and those reported previously for EMIM TFSI-based GPEs [14].

#### 2.1.2. ATR-FTIR Spectroscopy

ATR-FTIR spectra of PVdF-HFP films prepared using THF and NMP as solvents have been recorded. Once again there is a clear variation in the spectra. The spectrum of the PVdF-HFP film synthesized using THF shows bands assigned to the formation of a type α phase. However, when NMP is used as solvent, a different structure was obtained with bands attributed to a phase β of PVdF-HFP [3,6,14,30,31,32,33,34]. This has been comprehensively studied in reference 14.

**Figure 2:**
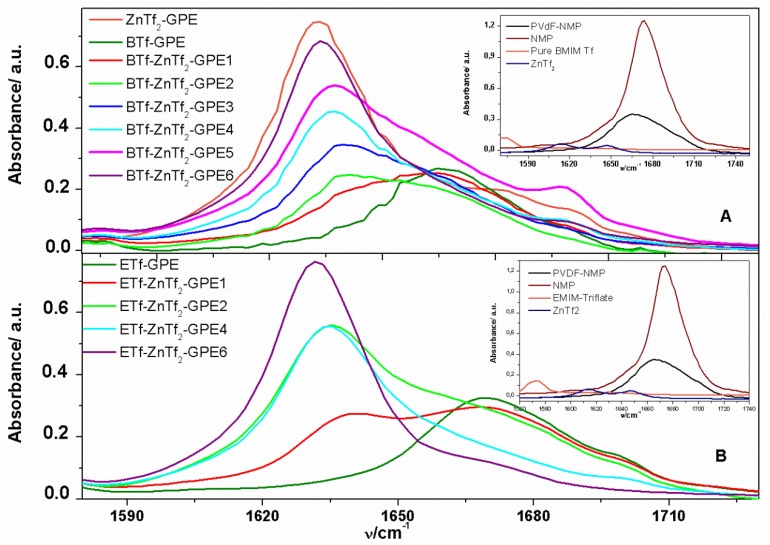
ATR-FTIR spectra of GPEs analyzed in the 1580–1730 cm^−1^ frequency range for (**A**) BTf-ZnTf_2._-GPEs; and (**B**) ETf-ZnTf_2_-GPEs. Insets: ATR-FTIR spectra of NMP solvent, pure IL, ZnTf_2_ GPE, and PVdF-HFP (NMP).

NMP characteristic bands can be observed in the ATR-FTIR spectrum of the PVdF-HFP(NMP) [14], among them there is an intense band at ~1667 cm^−1^, which has been attributed to the carbonyl group (Figure 2). These bands indicate that NMP molecules remain inside the polymeric matrix. Although the resulting position of the reflection can be influenced by many factors in such a complex system as this one, it is clear that the incorporation of NMP inside the polymer electrolyte increases the amorphous structure of the membrane, as it has been demonstrated by XRD spectra. Addition of IL and ZnTf_2_ salt to the PVdF-HFP prepared with NMP produced spectra with similar morphology, confirming that polar and amorphous structures were obtained [14].

NMP characteristic carbonyl band appearing at ~1667 cm^−1^ is shifted to minor wavenumber values, ~1637 cm^−1^, once ZnTf_2_ salt is incorporated into the membrane. Additionally, a rising intensity has been observed with the ZnTf_2_ concentration. This process is observed for ETf and BTf-based GPEs, coinciding with the behavior reported previously for ET-based GPEs [14]. Moreover, as can be seen in the insets in Figure 2, no shifts were observed when only ILs, without ZnTf_2_ salt, was incorporated inside the PVdF-HFP polymer. This fact suggests that the peak shifts are due to interaction between Zn^2+^ cations and the C=O groups of the NMP molecules. The ~1637 cm^−1^ band intensity was raised with the salt concentration, indicating that a higher quantity of Zn^2+^ cations interact with C=O groups of NMP until it reaches a maximum value (Figure 2). Similar observations have been explained as a consequence of the strong interaction between a EC plasticizer and lithium or zinc salts [12,13,14,35].

ZnTf_2_ has high dissociation ability due to the strong electron-withdrawing SO_2_CF_3_ groups and also has a low tendency to form ion-pairs [36]. Moreover, the high dielectric constant of PVdF-HFP (*ε* = 8.4) helps dissociating the salt and, hence, fostering high free ion concentration in the gel. This fact, together with a Zn^2+^-NMP interaction, should produce a strong dissociation of the ZnTf_2_, resulting in a great amount of free Tf anions. With the aim to confirm if Tf anions are free or forming ion pairs inside the GPE, we have analyzed the 1000–1080 cm^−1^ wavenumber range, where bands associated with free triflate anions (1030 cm^−1^), ion-pairs (~1042 cm^−1^), and higher aggregates (~1055 cm^−1^) formed by the triflate anions have been reported previously [35,37].

**Figure 3:**
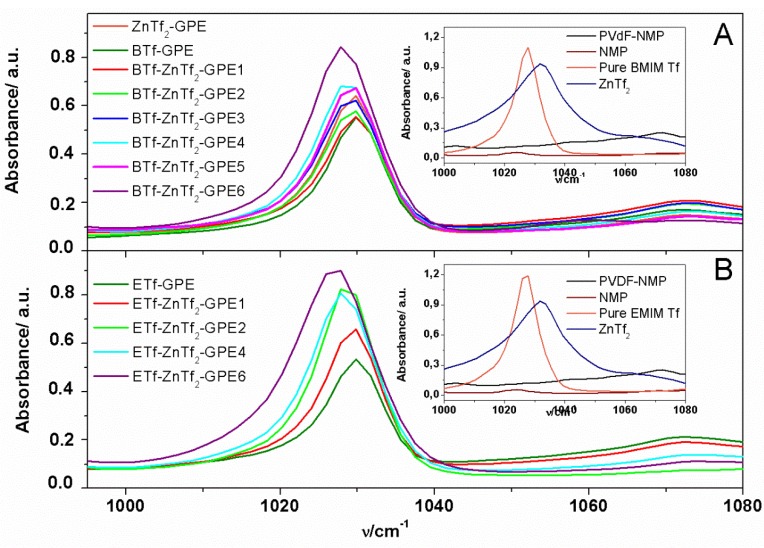
ATR-FTIR spectra of GPEs analyzed in the 1000–1080 cm^−1^ frequency range for (**A**) BTf-ZnTf_2._-GPEs; and (**B**) ETf-ZnTf_2_-GPEs. Insets: ATR-FTIR spectra of NMP solvent, pure IL, ZnTf_2_ GPE, and PVdF-HFP (NMP).

Figure 3 shows the 1000–1080 cm^−1^ range of the ATR-FTIR spectra obtained for ETf and BTf-based GPEs. As can be seen, only an intense band is obtained at ~1030 cm^−1^, indicating that free triflate anions are present for all IL-based GPEs. Furthermore, the peak intensity increases with the amount of salt added, which is indicative of the amount of free triflate anions increasing with the salt concentration. Moreover, this peak does not shift, confirming that a similar triflate interaction resulted for all GPEs prepared.

Regarding the other two triflate anion characteristic bands, due to ion-pairs (~1042 cm^−1^) and higher aggregates (~1055 cm^−1^) formation, they have not been detected. This behavior is in agreement with that observed previously for ET-based GPEs [14].

#### 2.1.3. Thermal Analysis

DSC analysis provides information on important parameters as glass temperature, melting point, and thermal stability of the GPEs. These parameters will affect the properties of the electrolyte material when operating in a battery. We have carried out a DSC study of the three different IL-based GPEs.

Glass transition temperature (Tg) is one of the most important parameters of the amorphous phase and it has been measured for all synthesized GPEs (Table 2). When only IL was added to the PVdF-HFP the resulting films produced the lowest Tg values, ~−80 °C, for the three IL-GPEs studied. However, inclusion of ZnTf_2_ without IL inside the GPEs provided the highest Tg value, ~−30 °C. Incorporation of IL and ZnTf_2_ together into the GPEs exhibited Tg intermediate values, ranging between −67 and −50 °C, which were shifted from lower to higher temperature with the increasing of the salt concentration. This result can be explained as due to that polymer chains movements are being hindered by the inclusion of a higher amount of salt. Additionally, Tg values are very similar for all GPEs analyzed, indicating that the degree of the hindrance should be similar regardless the IL type included in the GPE. This leads us to think that the interaction between polymer matrix and ILs are similar when enough amount of salt is added.

**Table 2:** Glass transition temperature, T_g_, ionic conductivity, σ, activation energy, E*_,_* and cationic transport number, t_+_, values for the gel polymer electrolytes studied. σ and t_+_ values obtained at 30 °C.

**GPE name**	**EMIM TFSI**	**EMIM Tf**	**BMIM Tf**	**EMIM TFSI**	**EMIM Tf**	**BMIM Tf**
**Sample**	**T_g_°C**			**σ(S cm^−1^)·10^−3^**		
IL-GPE	−81.6	−79.3	−78.8	1.98	7.07	5.2
IL-ZnTf_2_ GPE 1	−67.5	−64.8	−56.3	3.82	7.05	1.93
IL-ZnTf_2_ GPE 2	−58.5	−63.2	−56.3	3.73	4.62	2.68
IL-ZnTf_2_ GPE 3	−57.3	–	−55.3	3.05	–	2.99
IL-ZnTf_2_ GPE 4	−56.9	−61.1	−54.5	2.2	1.96	2.21
IL-ZnTf_2_ GPE 5	−53.3	–	−53.8	1.54	–	2.57
IL-ZnTf_2_ GPE 6	−49.4	−51.3	−51.1	1.88	1.09	1.88
**GPE name**	**EMIM TFSI**	**EMIM Tf**	**BMIM Tf**	**EMIM TFSI**	**EMIM Tf**	**BMIM Tf**
**Sample**	**t_+_**			**E_a_(eV)**		
IL-GPE	0.098	0.42	0.19	0.041	0.024	0.03
IL-ZnTf_2_ GPE 1	0.296	–	0.42	0.027	0.026	0.026
IL-ZnTf_2_ GPE 2	0.42	0.53	0.56	0.026	0.028	0.025
IL-ZnTf_2_ GPE 3	0.428	–	0.52	0.028	–	0.028
IL-ZnTf_2_ GPE 4	0.578	0.52	0.56	0.03	0.037	0.033
IL-ZnTf_2_ GPE 5	0.544	–	–	0.032	–	0.033
IL-ZnTf_2_ GPE 6	0.575	0.52	0.48	0.027	0.038	0.031

**Figure 4:**
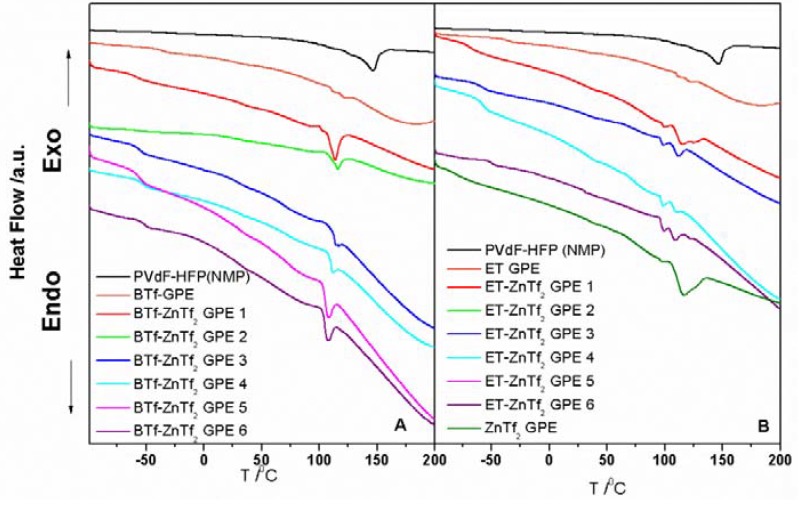
DSC plots of (**A**) BTf-ZnTf_2_ GPEs; (**B**) BTf-ZnTf_2_ GPEs.

In addition, endothermic peaks (Figure 4) related to the melting temperatures (Tm) have been observed in DSC measurements. For BTf-ZnTf_2_ GPEs Tm values show a very steady value of about 110 °C with a small trend to lower temperatures. A similar behavior is observed for ET-ZnTf_2_ GPEs, with Tm values also about 110 °C and same trend to lower temperatures.

Hence, Tg values shifting indicated that a large amount of the amorphous phase being present in the GPEs with the salt concentration, but it also suggest some restrictions in the flexibility imposed on the polymer chains [6]. From DSC analysis we can deduce that the type of anion or cation included in the IL does not induce important changes in the polymer matrix properties. Thus, the GPE’s behavior is mainly due to the polymer matrix, salt, and solvent interactions. Moreover, DSC curves demonstrate a stable thermal behavior in a wide range of temperatures, over 130 °C, which is vast enough to the application of these GPEs in batteries.

### 2.2. Electrical and Electrochemical Properties

2.2.1 Temperature Dependence of Ionic Conductivity

**Figure 5:**
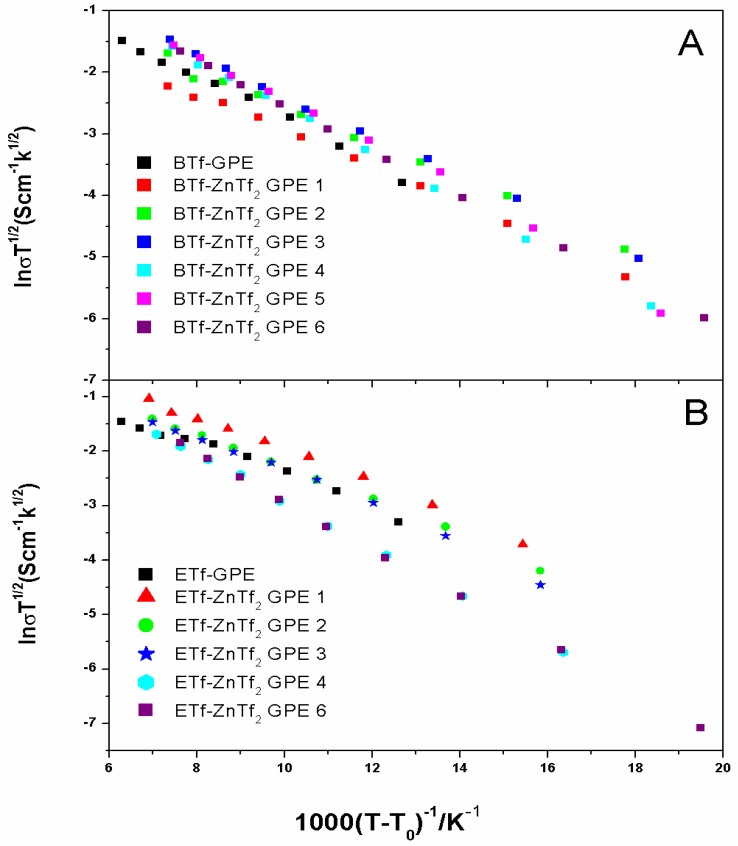
VTF plots of (**A**) BTf-ZnTf_2_ GPEs; and (**B**) BTf-ZnTf_2_ GPEs.

Figure 5 shows the variation of ionic conductivity with the temperature for ETf and BTf-based GPEs with different concentration of ZnTf_2_. All the plots obey the Vogel-Tammen-Fulcher (VTF) behavior throughout the temperature range under study, as it has been frequently observed for IL-based GPEs, and they are fitted to the equation:

(1) $$\sigma ={\text{AT}}^{-1/2}exp\left(-\frac{{\text{E}}_{a}}{{\;k}_{\text{B}}\left(\text{T}-{\text{T}}_{0}\right)}\right)$$

where A is the pre-exponential factor; E_a_ the activation energy; k_B_ the Boltzmann constant; T the testing temperature and T_0_ the equilibrium glass transition temperature. VTF behavior has been largely reported for IL-based polymer electrolytes [3,4,14,16,20,21,26].

From slopes in Figure 5 the activation energy values can be obtained, which are presented together with conductivity values for all ETf, BTf, and ET-based GPEs in Table 2. E*_a_* values for all GPEs ranged between 0.024 and 0.038 eV, and no relevant changes were observed for all GPEs, except for ET free ZnTf_2_ which have a maximum value of 0.041 eV.

Table 2 displays that all ionic conductivities obtained at 30 °C for the GPEs studied have similar values, ranging between 1.09 × 10^−3^ and 7.07 × 10^−3^ S·cm^−1^. However, note that ZnTf_2_ salt free GPEs provide very different values of the ionic conductivity: very low value for the ET-GPE, 1.5 × 10^−3^ S·cm^−1^, intermediate value, 5.3 × 10^−3^ S·cm^−1^, for BTf-GPE and the highest value, 7.07 × 10^−3^ S·cm^−1^, for ETf-GPE.

ZnTf_2_ addition produces different behavior for the three IL type GPEs. ETf-GPEs’ conductivity always decrease with the salt concentration, for ET-GPEs, the conductivity values increase initially with low salt quantity and, after that decrease, an addition of ZnTf_2_ to BTf-GPEs decrease the conductivity with respect to the salt-free GPE, but similar values are obtained in the whole concentration range.

From IL- ZnTf_2_ GPE 3 onwards we have obtained very similar conductivity values for each IL under same salt concentration, indicating clearly no significant variation or influence exerted by the IL anions or cations.

#### 2.2.2. Transport Number

**Figure 6:**
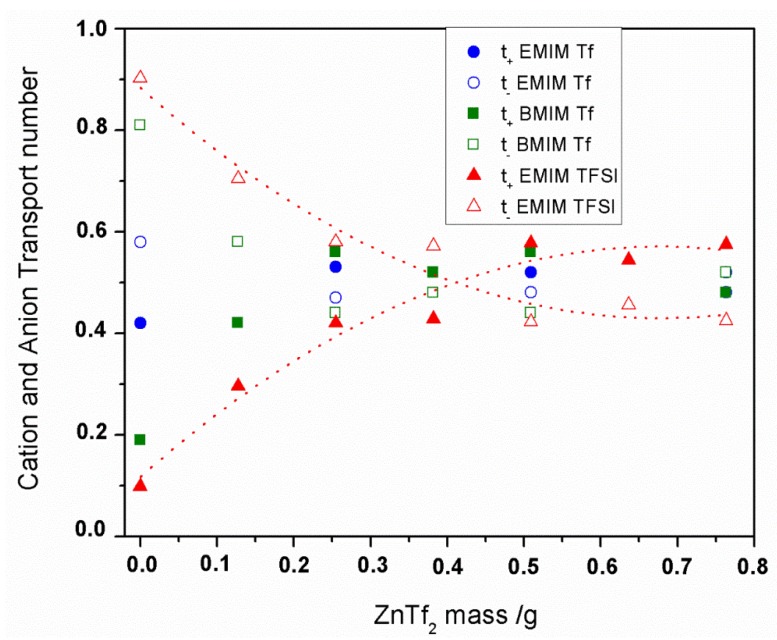
Cation and anion transport number for IL-ZnTf_2_ GPEs *vs* mass of ZnTf_2_ used. The legend indicates the IL names.

The total ionic transport number (t_ion_) was evaluated by the polarization technique [38], and values >0.98 were always obtained for the three IL-based GPEs under study, which indicates that the total conductivity was predominantly ionic. We have also determined the cationic transport numbers (t_+_) using the Evans method [14,22,23,39,40,41], because t_+_ values are a key factor in the optimization of electrolytes for zinc batteries. Figure 6 and Table 2 show the t_+_ values for the GPEs studied. Even if this method carries a certain degree of uncertainty, reproducible values were obtained within an acceptable error range. Three measurements were carried out to obtain each t_+_ value. Additionally, t- values are depicted in Figure 6.

We have analyzed how the transport cation number values are affected by varying the amount of salt inside the membrane. Figure 6 shows t_+_ and t_-_ values for the three-type IL-based GPEs. A similar tendency has been obtained for three kind IL-GPEs. As can be seen, at low salt concentrations into GPEs t- values are always higher than t_+_. However, t_+_ increases with the salt concentration inside the membrane for all GPEs, making t_+_ higher than t- for higher salt concentration GPEs.

Since EMIM^+^ and BMIM^+^ are bulky cations, mobility should be very low, and therefore the conductivity in these films will be predominantly anionic when salt have not been added yet to the system. Addition of ZnTf_2_ salt inside the GPEs provided higher t_+_ values with salt concentrations until it reaches a maximum value *ca.* 0.55 ± 0.05 for the three IL-based GPEs. These t_+_ values indicate that cation mobility increased when ZnTf_2_ salt was incorporated into the GPE, due mainly to Zn^2+^ takes over. Knowing that the EMIM^+^ is a bulky cations and BMIM^+^, even bulkier, mobility has to be very low due to great volume, as it was commented previously. Thus, we can consider that the cation transport values were due mainly to Zn^2+^ cations. Similar t_+_ values for the Zn^2+^ transport have been reported in the literature by different authors for several polymer electrolytes [4,14,41,42]. To this point, we have to consider the interaction between the Zn cations and carbonyl groups of the NMP molecules, as it was deduced from ATR-FTIR measurements. Recently, we have reported that ~1636 cm^−1^ peak intensity *versus* salt mass plot has a very similar tendency as the one obtained for t_+_ values, for ET- ZnTf_2_ GEP. Similar tendencies have been obtained for ETf- and BTf-based GPEs. Thus, we can deduce that the higher cationic transport numbers obtained for the GPEs with higher salt concentrations are a consequence of Zn^2+^ cations are solvated by NMP solvent, favoring the cation transport inside the GPE regardless the IL included in the GPE [14].

#### 2.2.3. Cyclic Voltammetry

**Figure 7:**
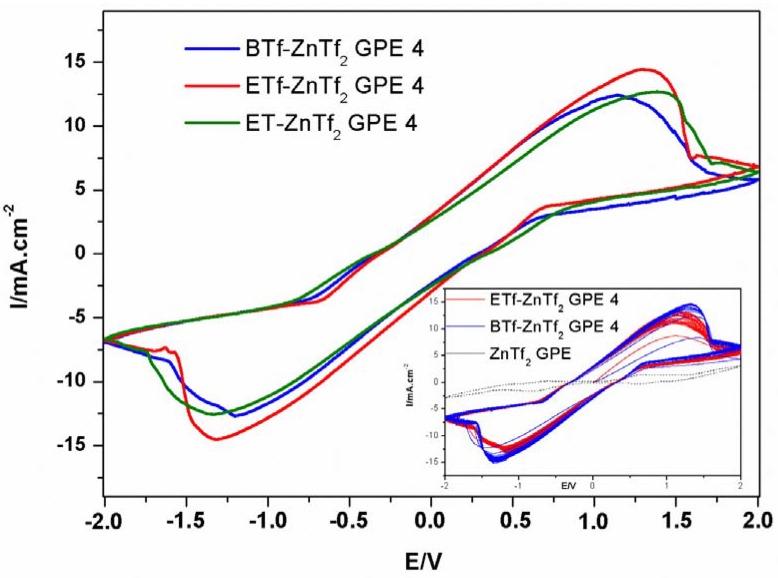
Cyclic voltammograms of Zn/GPE/Zn symmetric cells for BTf-ZnTf_2_ GPE 4, ETf-ZnTf_2_ GPE 4 and ET-ZnTf_2_ GPE 4. Inset: 20 consecutive cycles of ETf-ZnTf_2_ GPE 4 and BTf-ZnTf_2_ GPE 4 compared with ZnTf_2_-GPE voltammogram. Scan rate = 20 mV/s.

In order to confirm Zn^2+^ conduction into the GPEs, a cyclic voltammetric study was carried out using a Zn/GPE/Zn symmetric cell. Figure 7 shows the comparison of the voltammograms registered for three GPE types. No relevant differences were observed for all GPEs indicating that IL cation and anion types do not affect the general voltammetric behavior. We obtained similar density current values, *ca.* 15 mA·cm^−2^, and ∆E for the IL- ZnTf_2_ GPE 4. These current values are much higher than those obtained by us for similar GPEs using THF instead of NMP as solvent [3], confirming again the importance of the NMP molecules inside the membranes. Furthermore, reversibility of this system has been verified by the peaks stability observed when several consecutive cycles were registered for the GPEs (Inset in Figure 7 shows 20 consecutive voltammetric cycles for ETf-ZnTf_2_ GPE 4 and BTf-ZnTf_2_ GPE 4). Additionally, voltammograms corresponding to the ZnTf_2_-free IL-based membranes show that there is essentially no current for these GPEs [14]. No current was either obtained for IL-free membrane voltammograms, confirming that only ZnTf_2_ inside the polymer was not enough to produce good transport results. Finally, these voltammograms indicate that Zn^2+^ ions are indeed mobile in the GPEs and Zn is capable of dissolution into and deposition from the membranes, which is essential for their potential application in Zinc batteries [3,14,26]. Therefore, these results demonstrate that these membranes composed by PVdF-HFP, ZnTf_2_, and different ILs, such as EMIM Tf, BMIM Tf, or EMIM TFSI, may be used in Zinc-based batteries as GPEs [14,24].

#### 2.2.4. Zn/GPE/MnO_2_ Batteries

**Figure 8:**
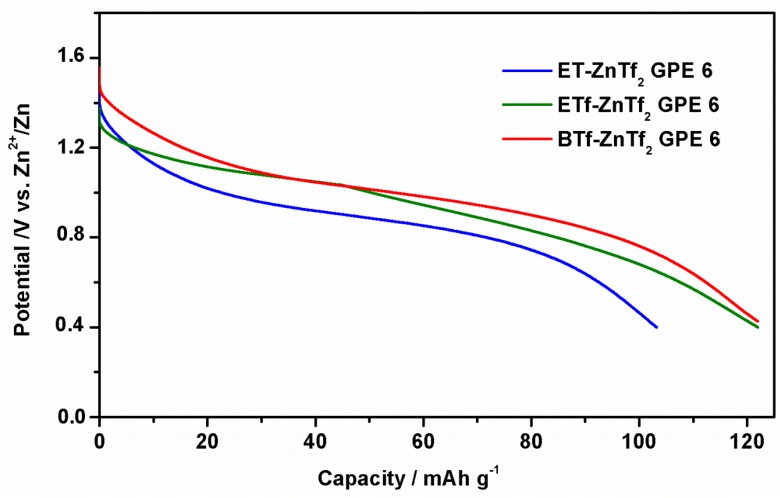
First discharge curves of a Zn/GPE/MnO_2_ cell at 1 mA·cm^−2^ for BTf- ZnTf_2_ GPE 6, ETf-ZnTf_2_ GPE 6, and ET-ZnTf_2_ GPE 6. Cut-off cell voltage was 0.4 V.

Finally, three ZnTf_2_-GPEs containing different ILs have been tested in a Zn/GPE/MnO_2_ battery at room temperature. Figure 8 shows the first discharge curve carried out at −1 mA·cm^−2^ to 0.4 V of the cut-off cell voltage, obtaining similar discharges capacities of 110–120 mAh·g^−1^. Once again no significant changes were found for different IL-based GPEs, confirming that IL type does no influence the GPE behavior.

### 2.3. Conductivity and ATR-FTIR Analysis of the IL-Based GPEs with and without ZnTf_2_

The results presented in this work point to a similar electrical and structural behavior for all IL-ZnTf_2_ GPEs synthesized, although three different ionic liquids were used. The three type GPEs show, when including an enough amount of ZnTf_2_ salt, similar ionic conductivity, activation energies, transport number, and Tg values. Furthermore, the voltammograms and discharge curves obtained for the GPEs synthesized with the same amount of ZnTf_2_ but changing the ionic liquid, present a very close behavior. In this sense, we wonder why the different cations and anions of the ionic liquids used have not affected the general behavior of the GPEs.

**Figure 9:**
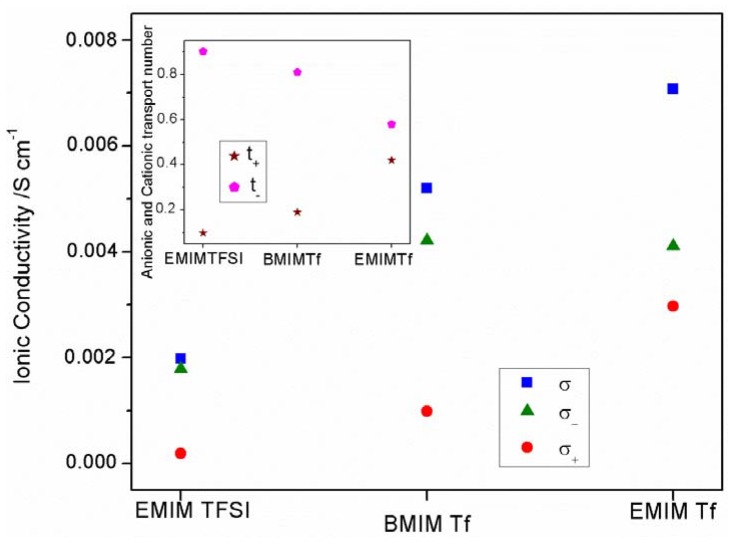
Total, σ, anionic, σ_-_, and cationic, σ_+_ conductivities for the three salt free IL-GPEs. Inset: Anionic and cationic transport number for the same salt free IL-GPEs.

With the aim to clarify this point, we have paid attention to the conductivity values obtained for the three salt-free GPEs (Figure 9). As can be seen, different conductivity values are obtained depending on the IL added, higher conductivity resulted in order ETf-GPE > BTf-GPE > ET-GPE. The ionic conductivity is very low for the ET-GPE, which contains TFSI^−^ anions; however, it is higher for ETf-GPE and BTf-GPE, both of them including Tf^−^ anions. This behavior seems to indicate that TFSI^−^ anion affects clearly the ionic conductivity of the ET-GPE, probably due to the interaction between TFSI^−^ and EMIM^+^, which will be very different to that resulting between BMIM^+^ or EMIM^+^ with Tf^−^.

To explain this behavior, additional analyses have been carried out. The conductivity, σ, can be expressed by [38,42]:

(2) $$\sigma =\sum^{\text{​}}n_{i}Z_{i}e\mu_{i}$$

where *n_i_* is the density of charge carriers of species *i*; *Z_ie_* the charge on these species and *u_i_* is the ionic mobility of species *i*. Ignoring the sign of the charge number *Z_i_*, we can write, for all 1:1 electrolytes:

(3) $$\sigma =e\left(n^{+}\mu^{+}+n^{-}\mu^{-}\right)$$

From this equation and considering the transport number measured for these GPEs, we can obtain the anionic, σ_-_, and cationic, σ_+_, conductivities as [22]:

(4) $$\sigma_{+}=\sigma \;t_{+}$$

(5) $$\sigma_{-}=\sigma \;t_{-}$$

Unfortunately, when more of two kinds of ions are including inside the GPEs, this model cannot be applied. Thus, to calculate the anionic, σ_-_, and cationic, σ_+_, conductivities for the GPEs containing IL and ZnTf_2_ we would have to know the charge carrier numbers and ion mobility values for all ionic species included in the GPE.

Figure 9 shows the total, anionic and cationic conductivities for the three salt-free GPEs. As can be seen, for all GPEs, σ_-_ is always higher than σ_+_, indicating that ion transport is mainly anionic. This result is in agreement with the t_+_ and t_-_ plot, inset in Figure 9. Furthermore, TFSI^−^ conductivity is lower than Tf^−^ conductivity observed for ETf and BTf GPEs.

This behavior may be explained in base of the different cationic-anionic interactions occurring in each GPE. In this sense, since the three ILs used in this work have an imidazolium cation, we have analyzed the 3000–3240 cm^−1^ region obtained by ATR-FTIR, where C-H imidazolium bands are presented.

**Figure 10:**
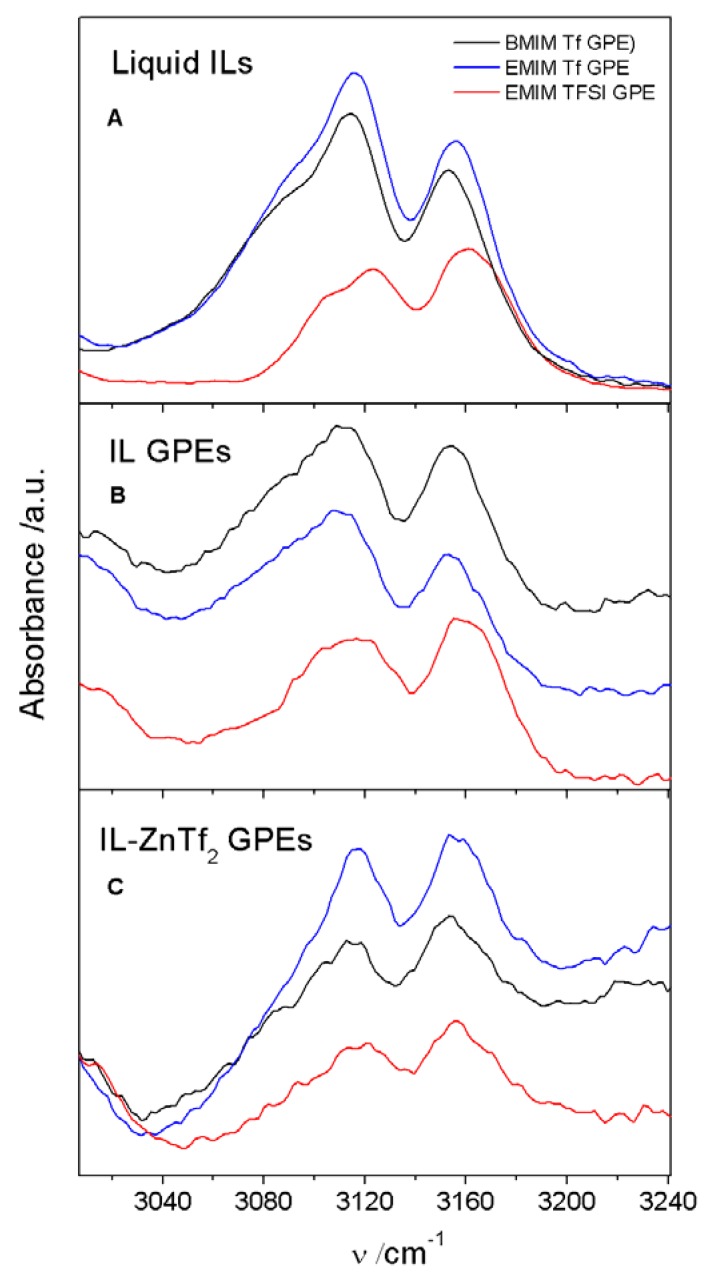
ATR-FTIR spectra at 3000–3240 cm^−1^ frequency range for (**A**) pure ILs; (**B**) salt free IL-GPEs; and (**C**) IL- ZnTf_2_ GPEs 4. The legend indicates the IL names.

In Figure 10, 3000–3240 cm^−1^ ATR-FTIR wavenumber range obtained for the three different ILs-based GPEs are compared. This figure includes the three salt free GPEs and IL-ZnTf_2_ GPE 4 membrane spectra. Additionally, ATR-FTIR spectra of pure ILs are included for comparison purposes. Figure 10A shows that pure EMIM TFSI bands have a shift with respect the other pure ILs. This shift is also observed for the salt free ET-GPE (Figure 10B). However, once the salt is included in the GPEs this shifting disappears (Figure 10C). The last result indicates that in these GPEs the IL cations will present very similar interaction with the corresponding anions.

**Figure 11:**
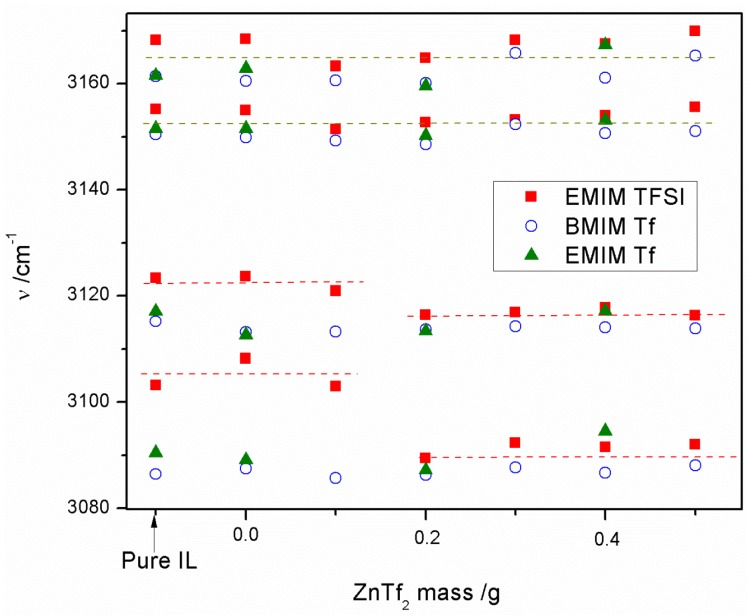
Deconvoluted peak wavenumbers from ATR-FTIR 3000–3240 range *vs*. mass of ZnTf_2_. The legend indicates ILs.

In order to highlight the magnitude of this shifting, we have carried out the deconvolution of ATR-FTIR bands and four peaks were obtained for all GPEs and pure ILs. Figure 11 displays the frequencies of the four ATR-FTIR bands for pure ILs and for all GPEs synthesized. This figure shows that no shifting is observed neither for the two highest frequency bands (~3165 and ~3155 cm^−1^) regardless the IL used, nor for pure ILs. Furthermore, both bands show similar and steady values for all GPEs throughout the whole salt concentration range. However, the other two bands (~3115 and ~3090 cm^−1^) are shifted to higher frequencies for pure EMIM TFSI IL and for free salt or low salt contain ET-GPEs. However, the rising of salt inside the GPEs produces that ET based GPEs show similar peak frequencies to those obtained for ETf and BTf-based GPEs. This result points to similar cation–anion interactions when enough concentration of ZnTf_2_ is added to the GPE.

## 3. Experimental Section

99% 1-Ethyl-3-methylimidazolium bis(trifluoromethylsulfonyl)imide (EMIM TFSI), 99% 1-Butyl-3-methylimidazolium trifluoromethanesulfonate (BMIM Tf), and 99% 1-Ethyl-3-methylimidazolium trifluoromethanesulfonate (EMIM Tf) were purchased from IOLITEC. The co-polymer poly(vinylidene fluoride-cohexafluoropropylene) (PVdF-HFP), tetrahydrofuran (THF), n-methyl-2-pyrrolidone (NMP), and zinc trifluoromethanesulfonate (ZnTf_2_) were obtained from Sigma-Aldrich (St. Louis, MO, USA). GPEs were prepared using a solution cast method reported previously [14]. Table 1 shows the weight of the components used in each prepared GPE.

X-ray diffraction patterns of the synthesized polymers were recorded using a Bruker (Billerica, MA, USA) D8-Advance Powder Diffractometer. ATR-FTIR spectra were obtained using a Thermo Nicolet 5700 Infrared Spectrometer (Thermo Fisher Scientific, Waltham, MA, USA) in the wave number range from 400 to 4000 cm^−1^. Ionic conductivity was determined from AC impedance measurements using a Biologic VSP Modular five-channel potensiostat/galvanostat in the frequency range from 100 kHz to 40 mHz. The temperature was set by a Julabo F25 thermostat (Julabo GMBH Seelbach, Germany) in the range from 278 to 353 K. The cell arrangement consisted of two platinum electrodes, which acted as blocking electrodes.

Ionic conductivity, σ, was calculated from the equation:

(6) $$\sigma =\frac{l}{R_{b}A}$$

where *l* is the thickness of the sample; *R_b_* the bulk resistance; and *A* the electrode/electrolyte contact area. The cationic transport number (t_+_) was evaluated using the Evans method [22,23]. AC impedance measurements were registered prior and after a DC polarization on the Zn/GPE/Zn cell. To polarize the cell a DC voltage (∆V = 0.05 V) was applied. t_+_ values were obtained from the next equation:

(7) $$t_{+}=\frac{I_{s}\left(\Delta V-R_{0}I_{0}\right)}{I_{0}\left(\Delta V-R_{s}I_{s}\right)}$$

where *I_0_* and *I_s_* are the initial and final currents and *R_0_* and *R_s_* are the cell resistances before and after polarization, respectively. Cyclic voltammetry was carried out using symmetric Zn/GPE/Zn cells.

GPEs were sandwiched between Pt and Pt electrodes (Zn/GPE/Pt cell) or two Zn discs (Zn(GPE/Zn cell). The contact area was always 0.5 cm^2^ and stainless steel current collectors were used. Zn discs (99.999%) and Pt plates (99.97%) used in these cells were purchased from Goodfellow (Huntingdon, England).

Galvanostatic charge/discharges were performed at 1 mA/cm^2^ using a Biologic VSP Modular five-channel potensiostat/galvanostat. Cathode materials were prepared mixing MnO_2_ (80%), carbon black (15%), and PVdF (5%) in 5 mL of THF. The slurry was stirred at room temperature for 1 h. After that, the slurry was dried in the oven at 80 °C for 1 h and finally was compacted at a pressure of 10 tons·cm^−2^. Thus, circular discs of diameter 12 mm were obtained. The specific capacity was calculated using the equation:

(8) $$C=\frac{I\;\times t}{m}$$

where *I* is the current constant intensity applied during the discharge process; *t* is the discharging time before to reach 0.4 V; and *m* is the total MnO_2_ active mass added in the cathode.

## 4. Conclusions

In view of the results we can postulate that a different structural and electrical GPEs behavior will be obtained when no salt or low salt concentration is included in the membrane, depending on the IL type used. However, when an enough amount of ZnTf_2_ salt is introduced in the GPE, very similar behavior resulted regardless the IL used. This result points to that the Zn^2+^ and Tf^−^ ion transport is predominant at high salt concentrations inside the GPEs. Additionally, when enough amount of ZnTf_2_ is included in the GPEs high cation transport number values were obtained, which has to be related with the NMP-Zn^2+^ interaction [14]. This fact makes Zn^2+^ the most important charge carrier in all GPEs regardless IL used. Thus, once again it is confirmed the relevance of the NMP presence inside the membrane to improve the conductivity properties of these GPEs.

In spite of this, inclusion of ILs inside the membranes is necessary to obtain GPEs with good electrical characteristics as it is demonstrated by the poor results obtained for IL-free GPEs in conductivities and VC measurements. A low ionic conductivity value of 1.1 × 10^−3^ S·cm^−1^ at 30 °C was obtained for IL free ZnTf_2_ GPE 2. Additionally, the inset in Figure 7 shows that VC peaks obtained for an IL free ZnTf_2_ GPE 2 are very smaller than those observed for BTf and ETf ZnTf_2_ GPEs. Consequently, ILs have an important role in the conductivity process inside the GPEs. Thus, though the inclusion of NMP is fundamental to raise the cation transport number, high-quality GPEs were only obtained when NMP, IL, and ZnTf_2_ were included into the polymer matrix.
